# The comparative role of cattle, goats and pigs in the epidemiology of livestock trypanosomiasis on the plateau of eastern Zambia

**DOI:** 10.1016/j.vetpar.2007.04.005

**Published:** 2007-07-20

**Authors:** H. Simukoko, T. Marcotty, I. Phiri, D. Geysen, J. Vercruysse, P. Van den Bossche

**Affiliations:** a University of Zambia, School of Veterinary Medicine, South Africa; b Institute of Tropical Medicine, Animal Health Department, Nationalestraat 155, B-2000 Antwerp, Belgium; c Ghent University, Vakgroep Virologie, parasitologie en Immunologie, Salisburylaan 133, B-9820 Merelbeke, Belgium; d Department of Veterinary Tropical Diseases, University of Pretoria, Onderstepoort, South Africa

**Keywords:** Trypanosomiasis, Livestock, Epidemiology, Zambia

## Abstract

To determine and compare the prevalence of trypanosome infections in different livestock species (cattle, pigs and goats) in areas where game animals are scarce and livestock constitute the main food source of tsetse, a survey was conducted on the plateau of the Eastern Province of Zambia in Katete and Petauke districts where *Glossina morsitans morsitans* is the only tsetse species present. Blood was collected from a total of 734 cattle, 333 goats and 324 pigs originating from 59 villages in both districts and was examined using the buffy coat method and the PCR-RFLP as diagnostic tools.

The prevalence of trypanosome infections differed substantially between livestock species. Using microscopic diagnostic methods, trypanosome infections were detected in 13.5% of the cattle and 0.9% of the pigs. All goats were parasitologically negative. The PCR-RFLP analyses increased the trypanosomiasis prevalence to 33.5, 6.5 and 3.3% in cattle, pigs and goats respectively. The majority of the infections (91.2%) were due to *Trypanosoma congolense*. The presence of a trypanosome infection in cattle and pigs resulted in a significant decline in the packed cell volume. The outcome of the study clearly shows that despite the availability of goats and pigs, cattle seem to be the major livestock species affected by the disease in trypanosomiasis endemic areas. The high proportion of infections in cattle could be partly attributed to their higher availability and attractiveness to tsetse.

## 1 Introduction

Tsetse-transmitted trypanosomiasis is an important constraint to livestock development in sub-Saharan Africa with estimated annual losses due to the direct and indirect consequences of the disease running into billions of dollars. About 10 million km^2^ of sub-Saharan Africa (representing about 46% of the total land) is infested with tsetse flies distributed over a belt spanning between latitudes 15°N and 20°S. Within this region, some 46–62 million head of cattle and other livestock species are at risk of the disease (Swallow, 1998).

The epidemiology of trypanosomiasis and its impact on livestock production varies from one locality to another and depends largely on the level of interaction between tsetse, domestic and game animals. The nature of that interaction is subject to spatial and temporal variations. Within the tsetse belts of southern Africa four distinct epidemiological situations can be distinguished: (i) wildlife zones where livestock is absent, (ii) areas where livestock have been recently introduced into wildlife zones, (iii) areas where livestock are present at the edge of wildlife zones (interfaces) and (iv) areas where livestock are kept in tsetse-infested zones and where large game animals are absent (Van den Bossche, 2001). Areas where livestock are kept in a tsetse-infested zone and where livestock constitutes the major host of tsetse are of particular economic importance. Such an epidemiological circumstance is usually the consequence of the gradual encroachment of people and their livestock into tsetse-infested areas and the subsequent disappearance of large game animals as a result of human interference and the clearing of vegetation for cultivation. It is found in large parts of the fertile and cultivated areas of southern Africa such as the plateau of the Eastern Province of Zambia. Since the mid-1940s, the plateau has been subject to human encroachment and large parts are currently cultivated. Cattle, goats and pigs are the main livestock species present. Game animals are scarce. *Glossina morsitans morsitans*, the only tsetse species present, is highly dependent on livestock for its survival (Van den Bossche and Staak, 1997). Although bovine trypanosomiasis is considered an important livestock disease on the plateau of eastern Zambia, little is known of the prevalence of trypanosome infections in pigs and goats. Nevertheless, pigs and goats can be suitable hosts for *G. m. morsitans* and are susceptible to infection with trypanosomes. To determine the relative importance of cattle, goats and pigs in the epidemiology of livestock trypanosomiasis on the eastern plateau of eastern Zambia and to assess the relevance of controlling the disease in livestock species other than cattle, a cross-sectional survey was conducted.

## 2 Materials and methods

### 2.1 Study area

The Eastern Province of Zambia lies between latitudes 10° and 15° S and longitude 30° and 33° east. It borders Malawi to the east and Mozambique to the south and covers an area of 69,000 km^2^, about 9% of Zambia's total territory. It is divided into eight districts: Chipata, Chama, Lundazi, Chadiza, Mambwe, Nyimba, Katete and Petauke. This study was conducted in the latter two districts. There are three distinct seasons: the warm wet season or agricultural season, from November to April; the cool dry season from May to August and the hot dry season from September to October. The average annual rainfall is about 1000 mm, with most of the rains occurring between December and March.

The plateau of the Eastern Province has a flat to gently rolling landscape with altitudes ranging from 900 to 1200 m. The vegetation is Miombo woodlands dominated by tree species such as *Braychystegia* and *Julbernadia* (Van den Bossche and De Deken, 2002). Most of the plateau is highly cultivated and carries a cattle population of approximately 11 animals/km^2^ in the settled areas (Doran and Van den Bossche, 1999). Maize, groundnuts and cotton are the main crops. A total of 63, 43 and 32% of the households in the study area own pigs, goats or cattle, respectively with an average number of 7 cattle, 5 goats and 5 pigs per owner (Doran, 2000). Goats and pigs roam freely in the vicinity of the villages. Cattle are usually herded but grazing patterns differ between seasons (Van den Bossche and De Deken, 2002). According to the 2003 livestock census, a total number of 49,089 cattle (Angoni breed), 24,211 goats (mainly Small East African breed) and 32,524 pigs were present in Katete district whereas 62,650 cattle, 39,565 goats and 39,142 pigs were present in Petauke district.

### 2.2 Sample selection

**Fig. 1:**
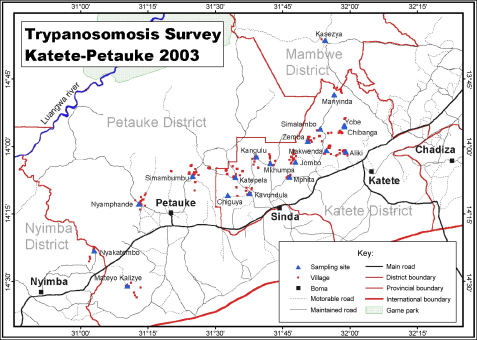
Location of sampling sites in Katete and Petauke districts of eastern Zambia.

The cross-sectional survey was conducted at 11 sampling sites (crushpens) in Katete and Petauke districts during the dry season (Fig. 1). The sample sizes at each of the sampling sites were calculated to provide 95% certainty of detecting at least one positive case at a prevalence of 5% (Cannon and Roe, 1982). The calculated sample size was 350 for each livestock species. A proportional stratified random sampling was applied to select cattle at each crushpen. Age and sex categories were considered as strata. Random sampling was then performed in such a way that the number of samples in each stratum was proportional to the herd structure described by Doran (2000). This was done to ensure that samples of all strata had the same weight. Villages within a perimeter of at least 2 km from where cattle were sampled were visited for goat and pig sampling. When sampling goats and pigs, a “home to home visit” sampling strategy was adopted in which all the goats or pigs from the homes were sampled to meet the required sample sizes.

### 2.3 Blood collection and diagnosis

From each selected animal, jugular blood was collected in a vacutainer tube with EDTA as anticoagulant. After sampling, the vacutainer tubes were placed in a cool box containing ice packs and transported to the laboratory within four hours of collection. From each vacutainer tube, blood was transferred into three capillary tubes which were sealed at one end with “Cristaseal” (Hawxley). The capillary tubes were spun in a microhaematocrit centrifuge for 5 min at 9000 rpm. After centrifugation, the packed cell volume (PCV) was determined. The buffy coat and the uppermost layer of red blood cells of each specimen were extruded onto a microscope slide and examined for the presence of motile trypanosomes. Samples were examined with a phase-contrast microscope at ×400 magnification (Murray et al., 1977). At least 50 fields were observed before declaring a slide as negative. Blood samples that were positive were further processed as blood smears for trypanosome species identification. Giemsa-stained thick and thin blood smears were examined under ×100 oil immersion objective lens (×1000 magnification).

The buffy coats of the two remaining capillary tubes were extruded onto a labelled filter paper (Whatman no 3, Whatman^®^). Filter papers were stored in sealed plastic bags containing silica gel and transferred in a freezer at−18 °C. The samples were further analysed using the PCR-RFLP described by Geysen et al. (2003). Briefly the protocol was as follows. Phenol extraction was used to extract the DNA from the filter papers. Standard PCR amplifications were carried out in 25-μl reaction mixtures containing 5-μl unknown sample, 50 mM KCl, 10 mM Tris–HCl (pH 8.3), 1.5 mM MgCl2, 200-μl of each dNTP, 20 pmol of each primer and 0.5 U Taq polymerase enzyme (Goldstar, Eurogentec). The reaction mixtures were placed on a heating block in a programmable thermocycler (Techgene, TECHNE DUXFORD, CAMBRIDGE, UK) with a heated lid. After a denaturation step of 4 min at 94 °C each of the 40 cycles consisted of 45 s at 92 °C, 45 s at 58 °C and 60 s at 72 °C. Semi-nested runs were performed in which 0.5-μl of amplification product from the first run was added to 24.5-μl of PCR mix at 84 °C (hot start principle), containing the same ingredients and concentrations except for 25 cycles. A negative control consisting of adding ultrapure water instead of template DNA to the PCR mixture was included in each PCR amplification. A 5-μl volume of each sample was electrophoresed in a 2% agarose gel for 20 min and stained with ethidium bromide for 30 min. A 100 bp DNA ladder (MBI Fermentas, Lithuania) was included in every gel. For further typing of the fragments, RFLP-based methods were used.

Primers used. The first amplification was done on the 18S gene using the forward primer 18ST nF2 (CAACGATGACACCCATGAATTGGGGA) and 18ST nR3 (TGCGCGACCAATAATTGCAATAC) as reverse primer. A semi-nested second amplification was done using the forward primer 18ST nF2 of the first amplification with the reverse primer 18ST nR2 (GTGTCTTGTTCTCACTGACATTGTAGTG).

RFLP-nested products were digested with *Msp* 1 and *Eco*571 enzymes in buffer Y + /Tango with *S*-adenosylmethionine according to the manufacturer's specifications (Gibco, UK) using 6-μl of amplified DNA in 15-μl total volume. The reaction was left overnight in a water bath at 37 °C. Four microlitres of restricted sample was then mixed with 2-μl loading buffer and transferred onto a 10% polyacrylamide gel together with a 100 bp DNA ladder (MBI Fermentas, Lithuania) for fragment size determination. DNA fragments were thereafter separated by horizontal electrophoresis in 0.5 × TBE buffer at 100 V for 2.5 h. The gels were stained using a commercial silver stain kit (Silver staining kit DNA plusone, Pharmacia Biotech, Uppsala, Sweden) and mounted for storage.

### 2.4 Statistical analysis

Statistical analyses were performed in Stata 9.1 (Sata Corp., 2003).

The prevalence of *Trypanosome* infections (determined using the PCR-RFLP) in the different species (cattle, goats and pigs) was compared in a single model. Explanatory variables were the crushpens, the host species and the interaction between the two. A poison regression specifying the exposure was applied since the prevalence was zero in a number of categories. Simplifications of the model were tested using the likelihood ratio test (cut off: *P* = 0.05).

For each of the three livestock species, the PCV data were analysed using linear regressions. The crushpens and the trypanosome infection status were used as categorical explanatory variables. The stratification of the cattle data was taken into account in the model.

Finally, the correlation between the prevalence of *T. congolense* infections in pigs and goats and its prevalence in cattle was analysed using a poisson regression specifying the exposure. The *T. congolense* prevalence in cattle of the respective sampling sites was used as continuous explanatory variable.

## 3 Results

A total of 734 cattle, 333 goats and 324 pigs originating from 59 villages surrounding the 11 sampling sites were sampled.

According to the results obtained using the microscopical diagnostic method, the proportion of infected cattle, goats and pigs was 13.5, 0 and 0.9, respectively (Table 1). Using the PCR-RFLP as diagnostic test, the proportion of infected cattle, goats and pigs was 33.5, 3.3 and 6.5%, respectively (Table 1). All parasitological positive animals were also positive on PCR-RFLP. In goats and pigs, all infections were due to *T. congolense*, whereas in cattle, the majority of the infections (91.2%) were due to this trypanosome species (Table 1). The *T. congolense* prevalence of infection, determined using the PCR-RFLP, differed substantially between sampling sites with the highest proportion of infections in animals sampled at sites located in Katete district (Table 2). According to the statistical analyses, cattle were significantly more infected with *T. congolense* than goats and pigs (*P* < 0.001 for both). The prevalence of trypanosome infections in goats and pigs did not differ significantly (*P* < 0.28). The proportion of infected cattle at a sampling site was significantly (*P* < 0.03) correlated with the proportion of trypanosome infections in pigs but not in goats (*P* < 0.365). The proportion of infected oxen, cows, young males, young females and calves is summarised in Table 3. The differences in the proportion of infected animals belonging to the various age categories and the sexes were statistically not significant (*P* = 0.44%).

The presence of a trypanosome infection in cattle, pigs or goats significantly reduced the PCV. The PCV of infected cattle was on average 8.5% lower (*P* < 0.001) compared to the PCV of non-infected cattle (Table 4). For infected goats and pigs, on the other hand, trypanosome infection reduced the PCV on average by 4.8 and 13.6%, respectively (*P* < 0.001 for both goats and pigs).

## 4 Discussion

Hitherto, parasitological diagnostic methods and the antibody detection ELISA have been used extensively for epidemiological studies in the Eastern Province of Zambia (Sinyangwe et al., 2004; Van den Bossche et al., 2004; Machila et al., 2001; Van den Bossche and Rowlands, 2001). The parasitological prevalence of bovine trypanosomiasis observed using the buffy coat method in this study was similar to the prevalence observed during other surveys but was substantially lower than the prevalence determined using a molecular diagnostic tool. This is not surprising considering the low sensitivity of parasitological diagnostic methods (Picozzi et al., 2002). This is especially so when the parasitaemia is low and may explain the very low parasitological prevalence of trypanosome infections in goats and pigs (MacLennan, 1970; Omeke, 1994). The antibody detection ELISA, on the other hand, has high sensitivity but detects antibodies against current and past infections (Van den Bossche et al., 2000). Hence, the antibody detection ELISA overestimates the actual prevalence of infection. Although the sensitivity of molecular diagnostic tools such as the PCR-RFLP is also affected by the parasitaemia; the outcome of the molecular diagnosis is probably a good representation of the proportion of animals of each of the main livestock species present in the study area that are infected with trypanosomes. The high proportion of *T. congolense* infections is in accordance with observations made in other southern African countries (Van den Bossche, 2001). The absence of *T. vivax* infections in goats is attributed to the overall low prevalence of trypanosome infections in this livestock species. Since *T. vivax* is not infective for pigs, none of the pigs were infected with this trypanosome species. The high trypanosomiasis prevalence in Katete district compared to Petauke district is attributed to higher level of cultivation and vegetation clearing resulting in a substantial destruction of suitable tsetse habitat in the latter district.

The probability of a host contracting trypanosomiasis depends on the rate at which it is fed upon by infected tsetse flies (Rogers, 1988). The attraction of tsetse flies to a host and subsequently the proportion of tsetse that feed and challenge that host is the result of a number of stimuli. The number of tsetse attracted to a host is determined largely by the amount of odour (mainly carbon dioxide and other unidentified kairomones) produced by that host or, in the case of a herd of potential hosts, by that herd (Torr et al., 2006). The probability that an attracted tsetse fly feeds on that host is determined by short-range visual (Vale, 1974) and olfactory stimuli (Warnes, 1995) and the behaviour of the host (Torr and Mangwiro, 2000). Hence, host preference and thus challenge is the result of a range of factors that may differ depending on the ecological conditions. Under the conditions prevailing on the plateau of eastern Zambia where livestock constitutes the potential hosts of tsetse, cattle are the preferred host and undergo the highest level of challenge. There are a number of reasons why cattle are the most preferred tsetse host in the Eastern Province. First, cattle are spread more evenly in the study area and are thus more available, whereas the distribution of goats and pigs is restricted to the vicinity of villages (Van den Bossche and De Deken, 2002). Second, because of the odour plumes produced by individual cattle and the large odour plumes produced by cattle grouped in herds, tsetse flies are expected to be far more attracted to cattle than pigs or goats. Similar low levels of challenge of goats have been observed in other tsetse-infested areas of Zambia (Ahmadu et al., 2002). Nevertheless, goats can be an important tsetse host and become readily infected with trypanosomes. This was, for example, the case in the Luangwa Valley of eastern Zambia where the incidence of trypanosome infections in goats was high with a significant impact on goat production (Bealby et al., 1996). It thus seems that despite the defensive behaviour of goats and in the absence of alternative more attractive hosts, goats can be fed upon frequently. Although suidae are considered preferred hosts of *G. m. morsitans* (Clausen et al., 1998; Torr, 1994; Weitz, 1963), the prevalence of trypanosome infections in domestic pigs was relatively low. This is again attributed to the higher availability and attractiveness of cattle and the restricted distribution of pigs. Nevertheless, the relationship between the proportion of infected cattle and the proportion of infected pigs suggest that when challenge is high, pigs are more readily fed upon. This may indicate a density-dependent feeding success on cattle which has already been suggested by Vale (1977). According to our results, it thus seems that in an area where livestock constitute the main host of tsetse, cattle act as a protective shield by attracting the majority of the tsetse flies and protecting other livestock species from high levels of tsetse challenge. Hence, in the presence of cattle, trypanosomiasis seems to be of minor importance in other livestock species. Nevertheless, despite the low proportion of infected pigs, the infection caused severe anaemia in this livestock species. Such high levels of anaemia in pigs infected with *T. congolense* have been reported elsewhere (Omeke, 1994). In goats, on the other hand, the average PCV of infected animals was only 4.8% lower suggesting a level of tolerance. In cattle, trypanosome infections caused a variable level of anaemia. Such variations between cattle herds or cattle sampled at various sampling points are attributed mainly to differences in disease management practices and possibly differences in the virulence of circulating trypanosome strains (Masumu et al., 2006).

Because of human encroachment into tsetse-infested areas, the importance of livestock as host of tsetse flies is likely to increase substantially. The repercussions of this change in host preference on livestock production and productivity will depend largely on the livestock species present and their attractiveness to tsetse flies. Depending on the circumstances, the role of small ruminants and pigs in the epidemiology of livestock trypanosomiasis may be minimal. In the absence of cattle, on the other hand, pigs and goats can be suitable hosts and challenge may impact substantially on their productivity. In such situations, control of the disease in those species is advisable.

## Figures and Tables

**Table 1:** Observed proportions (±S.D.) of cattle, goats or pigs infected with trypanosomes using the PCR-RFLP or parasitological diagnostic

	*n*	PCR-RFLP				Parasitology			
		*T. congolense* (%)	*T. vivax* (%)	Mixed^a^ (%)	Total^b^ (%)	*T. congolense* (%)	*T. vivax* (%)	Mixed^a^ (%)	Total^b^ (%)
Cattle	734	30.5 ± 1.7	2.4 ± 0.6	0.5 ± 0.3	33.5 ± 1.7	10.6 ± 1.1	2.2 ± 0.5	0.7 ± 0.3	13.5 ± 1.3
Goats	333	3.3 ± 1	0	0	3.3 ± 1	0	0	0	0
Pigs	324	6.5 ± 1.4	0	0	6.5 ± 1.4	0.9 ± 0.5	0	0	0.9 ± 1.6

a *T. congolense* and *T. vivax.*

b *T. congolense*, *T. vivax* and mixed.

**Table 2:** Observed and predicted (CI) proportions of cattle, goats, or pigs infected with *T. congolense* at 11 sampling sites on the plateau of eastern Zambia

Sampling site	Cattle			Goats			Pigs		
	*n*	Observed (%)	Predicted (%)	*n*	Observed (%)	Predicted (%)	*n*	Observed (%)	Predicted (%)
Alick (K)	77	31.2	27.6 (18.5–41.3)	35	2.9	3.1 (1.5–6.2)	36	8.3	4.6 (2.6–8.2)
Chiguya (K)	68	30.9	27.0 (17.6–41.6)	35	5.7	3.0 (1.5–6.1)	35	2.9	4.5 (2.5–8.1)
Makwenda (K)	83	53	47.1 (35.0–63.3)	31	9.7	5.2 (2.7–10.0)	55	1.8	7.8 (4.7–12.8)
Katepela (K)	60	78.3	68.8 (51.5–92.0)	45	2.2	7.6 (4.0–14.3)	20	20	11.3 (6.8–19.1)
Nyakatembo (P)	57	1.8	4.5 (1.4–14.0)	54	3.7	0.5 (0.1–1.7)	23	0	0.7 (0.2–2.5)
Manyinda (K)	61	47.5	46.0 (32.7–64.5)	34	2.9	5.1 (2.6–10.0)	69	11.6	7.6 (4.6–12.6)
Kasero (P)	59	1.7	1.6 (0.2–11.2)	39	0	0.1 (0.02–1.3)	0		
Nyamphande (P)	66	1.5	0	0			0		
Jombo (K)	70	44.3	39.6 (27.8–56.5)	22	4.5	4.4 (2.2–8.7)	35	5.7	6.6 (3.8–11.3)
Simabumbu (P)	62	9.8	8.9 (4–19.7)	29	0	1.0 (0.3–2.6)	15	0	1.5 (0.6–3.6)
Zemba (K)	71	62	55.1 (40.8–74.5)	9	11	6.1 (3.1–11.9)	36	5.6	9.1 (5.5–15.2)

(K): Katete; (P): Petauke.

**Table 3:** Proportions of cattle belonging to different categories infected with *T. congolense* and sampled at 11 sites on the plateau of eastern

Sampling site	*n*	Age/sex category										
		Calves	*n*	Young females	*n*	Young males	*n*	Cows	*n*	Bulls	*n*	Oxen
Alick (K)	9	0	4	50	0		28	32	3	33	33	36 4
Chiguya (K)	23	22	6	17	4	0	16	31	0		19	53
Makwenda (K)	5	0	11	36	0		29	62	3	67	35	57
Katepela (K)	1	0	1	0	5	100	20	70	0		33	82
Nyakatembo (P)	3	0	4	0	2	0	17	0	0		31	3
Manyinda (K)	4	50	8	12	0		19	53	3	33	27	56
Kasero (P)	22	0	0		1	100	6	0	0		30	0
Nyamphande (P)	0		0		0		34	3	0		32	0
Jombo (K)	5	0	10	20	9	22	21	67	0		25	52
Simabumbu (P)	8	12	2	0	6	17	21	10	1	0	24	80
Zemba (K)	4	75	6	0	13	77	17	53	3	33	28	54

Total	84		52		40		228		13		317	

**Table 4:** Observed and predicted (CI) PCVs of infected and non-infected cattle on the plateau of eastern Zambia

Sampling site	Infected			Non-infected	
	*n*	Observed	Predicted (CI)	Observed	Predicted (CI)
Alick (K)	77	24.1	23.5 (22.5–24.6)	32.1	32.0 (31.1–33.0)
Chiguya (K)	68	24.2	22.6 (21.5–24.0)	31.1	31.1 (30.1–32.2)
Makwenda (K)	83	21.7	22.2 (21.2–23.3)	31.4	30.8 (29.7–31.8)
Katepela (K)	60	23.7	23.4 (22.0–25)	32.1	31.8 (30.3–33.4)
Nyakatembo (P)	57	28	24.7 (23.3–26.1)	33.1	33.2 (32–34.3)
Manyinda (K)	61	22.1	21.8 (21.0–22.8)	30.1	30.3 (29.4–31.3)
Kasero (P)	59	25	22.2 (20.8–23.5)	30.6	30.6 (29.6–31.7)
Nyamphande (P)	66	20	21.7 (20.4–23)	30.2	30.2 (29.2–31.3)
Jombo (K)	70	23.9	24.2 (23.1–25.4)	32.9	32.7 (31.7–33.7)
Simabumbu (P)	62	23	25.5 (24.1–26.9)	34.2	34 (32.7–35.2)
Zemba (K)	71	22.8	22.6 (21.6–23.5)	31.2	31.1 (30.0–32.1)

(K): Katete; (P): Petauke.
